# Effects of Vitamin D Treatment on Skeletal Muscle Histology and Ultrastructural Changes in a Rodent Model

**DOI:** 10.3390/molecules17089081

**Published:** 2012-07-31

**Authors:** Khalid M. Alkharfy, Nasser M. Al-Daghri, Mukhtar Ahmed, Sobhy M. Yakout

**Affiliations:** 1 Department of Clinical Pharmacy, College of Pharmacy, King Saud University, Riyadh 11451, Saudi Arabia; 2 Prince Mutaib Chair for Osteoporosis, King Saud University, Riyadh 11451, Saudi Arabia; 3 Biochemistry Department, College of Science, King Saud University, Riyadh 11451, Saudi Arabia; 4 Transmission Electron Microscope Unit, College of Science Research Centre, King Saud University, Riyadh 11451, Saudi Arabia

**Keywords:** calcitriol, skeletal muscles, histopathology, ultrastructural changes

## Abstract

Vitamin D is well known for its role in maintaining calcium and phosphorus homeostasis and in promoting bone mineralization; however, more of its pleiotropic effects have been described recently. The aim of the present investigation was to study the effect of vitamin D treatment on skeletal muscles changes under different dietary conditions using an animal model. Four groups of C57BL/6J mice (n = 11 each) were maintained on either low fat diet (LFD) or high fat diet ‎‎(HFD) with and without 1α,25–dihydroxyvitamin D3 (calcitriol) for 16 weeks. Animal weigh was recorded at baseline and then regular intervals, and at the end of the study, skeletal muscle tissues were harvested for the evaluation of the histopathological and ultrastructural changes. When control C57BL/6J mice were fed high-fat diet for 12 weeks, body weight gain was significantly increased compared with mice fed a LFD. (30.2% *vs.* 8.4%, *p <* 0.01). There was a significant gradual decrease in the weight of HFD fed mice that were treated with ‎vitamin D as compared with a steady increase in the weights of controls (6.8% *vs.* 28.7%, *p <* 0.01). While the LFD group showed some ultrastructural changes, HDF fed on mice showed great muscle structural abnormalities. The whole sarcosome along with its membrane and cristae were severely damaged with scattered myocytes in HFD group. Furthermore, the mitochondria appeared weak and were on the verge of degenerations. The bands were diminished with loss of connections among myofibrils. These changes were attenuated in the HFD group treated with vitamin D with tissues have regained their normal structural appearance. ‎The current findings indicate an important effect of vitamin D on skeletal muscle histology under HFD conditions.

## 1. Introduction

Skeletal muscle is a heterogeneous tissue made up of different fiber types, in which glycolysis and mitochondrial oxidative phosphorylation for energy production takes place [1]. Skeletal muscle oxidative capacity is determined by mitochondrial biogenesis [2], which involves both proliferation and differentiation processes [3]. Mitochondrial dysfunction involved in alteration of oxidative metabolism is thought to play a crucial role ‎ in insulin resistance [4]. ‎However, the cause-and-effect of this relationship between mitochondrial dysfunction and the development of diabetes remains unclear [4,5,6].

Obesity may be characterized by fat accumulation in skeletal muscle, and this changes likely leads to long-term metabolic derangements including type 2 diabetes mellitus (T2DM) [7]. Insulin resistance, a whole mark of T2DM, is caused by the ‎‎inability of insulin-target tissues to respond properly to insulin [8], and in whose ‎‎etiology mitochondrial dysfunction is thought to play a crucial role [4,5]. ‎In addition, visceral fat depots have direct portal access, and thus, a greater potential to harm the liver, impaired glucose uptake by skeletal muscle and increased basal lipolytic rate and free fatty acid release. 

Vitamin D deficiency has been associated with ‎diabetes mellitus and its ‎correction ‎contributes to decreased diabetes risk and cardiovascular ‎diseases, underlying the ‎importance of ‎its role in the prevention of several non-communicable ‎diseases [9]. ‎‎Increasing evidence also indicates that vitamin D plays an essential role in many tissues including skeletal muscle. Early clinical descriptions of a myopathy associated with severe vitamin D deﬁciency indicated a potential association between vitamin D and muscle [10]. Indeed, skeletal symptoms were found to be responsive to treatment with vitamin D; however, the underlying mechanisms remained undeﬁned [11]. Taken together, the present study sought to elucidate the effect of vitamin D on histopathological and ultrastructural changes in skeletal muscle in a rodent model fed on high and low fat diet. 

## 2. Results

Exposure ‎to HFD lead to significant body weight gain as compared with that of LFD groups by weeks 8 and 12 where the mean percentage weight gain were ‎‎26.4% *vs.* 6.4% (*p <* 0.05) and 30.2% *vs.* 8.4% (*p <* 0.01), respectively. The body weight of HFD ‎fed animals became diﬀerent as the weight of mice gradually but noticeably decreased in vitamin D ‎group compared with its control group after 12 weeks of treatment (11.4% *vs.* 30.2%, *p <* 0.05). At the ‎end of the study, those animals which received HFD with vitamin D had the smallest percentage weight ‎gain as opposed to controls (6.8% *vs.* 28.7%, *p <* 0.01).

**Figure 1:**
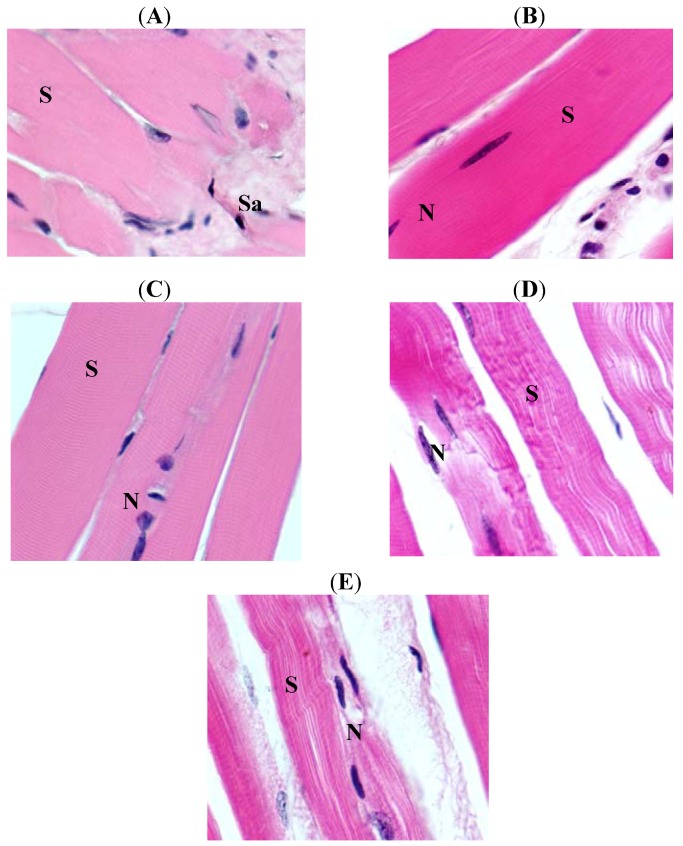
Histology of muscle samples. (**A**) Interstitial fibrosis changes in skeletal muscle (×1,000) of Group I. (**B**) Skeletal muscles with normal architecture (×1,000) of Group II. (**C**) Skeletal muscles with normal architecture (×1,000) of Group III. (**D**) Skeletal muscle with wavy appearance fibers (×1,000) of Group IV. (**E**) Normal architecture of muscle fibers (×1,000) of Control.

**Figure 2:**
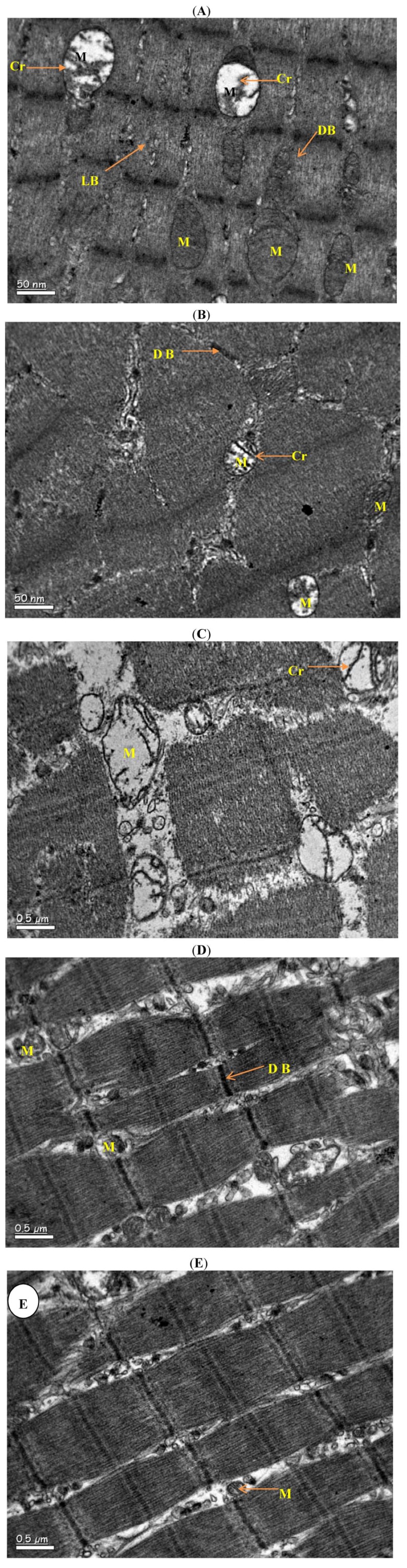
Electron photomicrographs of muscle samples. (**A**) Group I, Normal general ultrastructural appearance of skeletal muscle, note damaged cristae of mitochondria (20,000×). (**B**) Group II, Normal general ultrastructural appearance of skeletal muscle, note normal structure of mitochondria (20,000×). (**C**) Group III,‎ Different in size and damaged cristae of mitochondria within an intermyofibrillar space (20,000×). (**D**) Group IV, Normal general ultrastructural appearance of skeletal muscle, with focal edema within an intermyofibrillar space (20,000×). (**E**) Control, Normal general ultrastructural appearance of skeletal muscle, with normal structure of mitochondria(20,000×).

Skeletal muscle of mice fed on LFD showed a disturbance in sarcosome, sarcosomal membrane and sarcosomal cristae. There was accumulation of some fussy materials. The remaining structural is intact with other normal features (Figure 1A and Figure 2A). Vitamin D treatment was associated with normalization of the LFD induced skeletal muscle and is normal as in controls (Figure 1B and Figure 2B). However, HFD treatment induced a great disturbance in sarcosome region, the whole sarcosome along with its membrane and cristae are severely damaged. The mitochondria were on the verge of degenerations. There was also accumulation of fussy materials in sarcoplasm; whereas sarcolemma and sarcomere were well disturbed with scattered myocytes. The bands are diminished with loss of connections among myofibrils (Figure 1C and Figure 2C). Vitamin D treatment of mice feed on HFD was associated with normalization of skeletal muscle features (Figure 1D and Figure 2D), which were similar to that of untreated controls (Figure 1E and Figure 2E).

## 3. Discussion

This study was undertaken to evaluate the effect of vitamin D treatment on muscle histological and ultrastructural changes associated with weight gain in a C57BL/6J mouse model. This mouse strain has especially been used as a human obesity model because it develops obesity, insulin resistance and hyperlipidemia when raised on a high-fat and high-sucrose diet; however, it remains lean if the fat content of the diet is limited [12,13,14,15].

Skeletal muscle oxidative capacity is determined by mitochondrial function and biogenesis [2]. Indeed, it is well established that mitochondria takes active part in aerobic biogenesis and oxidation of skeletal muscles [16]. Mitochondrial biogenesis is involved in the proliferation and differentiation of mitochondrial number and an improvement of the functional capabilities of pre-existing mitochondria [3]. Therefore, mitochondrial dysfunction has been proposed to be involved in the alteration of oxidative metabolism associated to insulin resistance [17]. However, the cause-and-effect relationship between mitochondrial dysfunction and the development of insulin resistance remains unclear [4,5,6]. In the present study, a large number of mitochondria were in the process of degeneration, which was observed in mice fed on either LFD or HFD. This in turn can affect the oxidation capacity and the biogenesis process.

Even though vitamin D deficiency has long been associated with muscle weakness [18,19], until recently no etiological mechanism has been described. Vitamin D treatment has been shown to be protective against the development of insulin resistance in mice [20]. The evident effect of vitamin D treatment on the histological abnormalities of the muscles in the LFD and HFD group also accentuates its role as a muscular-protective agent [21]. To our knowledge, the current data is the first to determine a causal relationship between vitamin D intake and mitochondrial degeneration in myocytes under high fat fed conditions using an animal model. This effect is possibly mediated through a modulation in the circulating levels of adipokines. For example, adiponectin is a hormone secreted by adipocytes that plays an important role in the regulation of mitochondrial biogenesis and insulin sensitivity [1,22,23]. Adiponectin binds to its receptors (AdipoR1, the most abundantly expressed in skeletal muscle, and AdipoR2) activating 5'-AMP-activated protein kinase (AMPK), which finally leads to the stimulation of glucose uptake and fatty acid oxidation [22,23].

## 4. Experimental

### 4.1. Animals and Study Protocol

Male C57BL/6J mice aged 4–5 weeks and weighing 20–25 g were obtained from Animal House Care Center, College of Pharmacy, King Saud University, Riyadh, Saudi Arabia. The Ethics Committee of the Experimental Animal Care Center, College of Pharmacy, King Saud University, Riyadh, Saudi Arabia, approved the conduct of experiments. All animal were housed in a temperature controlled room on a 12 h light/dark cycle and had free access to water and normal chow *ad libitum*. The mice were allowed to acclimatize for one week before being introduced into the study. After conditioning, mice were randomly divided into four groups of eleven animals each in each group. Group I was a control group fed a 10 kcal% (Fat 4.3 g %) low fat diet ‘LFD’ (Research Diets Inc., New Brunswick, NJ, USA) and coconut oil (1 mL/day). Group II was fed LFD and received calcitriol (1,25-(OH)_2_D_3_, (Rocaltrol^®^, Hoffman-LaRoche Ltd, Basel, Switzerland) diluted with coconut oil and given at a dose of 150 IU/kg/day by oral gavage (1 mL) [24] Group III served as a control group and received 1 mL coconut oil daily and supplied with 45 kcal% (fat 24 g %) high fat diet ‘HFD’ (Research Diets Inc., New Brunswick, NJ, USA). Group IV was an experimental group fed on HFD and treated with 150 IU/kg/day calcitriol orally (delivered as 1 mL in coconut oil by oral gavages). Treatment was carried out for 16 consecutive weeks and animal weight was recorded at baseline and monthly thereafter. A separate control, without any treatment was also kept to support the histopathological evaluation.

### 4.2. Histology and Transmission Electronic Microscope ‎Studies

At the end of the experiment, mice were anesthetized with ether and immediately euthanized by ‎cervical dislocation.‎ The skeletal muscle tissue were removed and fixed in 10% buffer saline and was processed to get paraffin sections ‎(4–5 µm) ‎for the histological study using hematoxylin and eosin stain (Drury and Wallington 1967) to be examined under light microscopy. For Transmission Electronic Microscope ‎(TEM) evaluation, small muscle pieces were cut (~2 mm) and fixed in 3% gluteraldehyde for 4 h and washed in 0.2 M sodium cacodylate buffered saline (pH 7.4). Post fixation was performed with 1% osmium tetra-oxide for 1 hour, and then tissues were washed in phosphate buffered saline and dehydrated in alcohol (50%, 70%, 80%, 95%, and 100%). Tissues were further treated with propylene oxide (for 30 min), propylene oxide-resin mixture (overnight), and pure resin (for 48 h). Embedding was done in BEEM (better equipment for electron microscopy) capsules using pure Spurr’s low viscosity resin at 80 °C for 48 h. Ultrathin sections (70 nm) were taken using Leica EM UC 6 ultramicrotom ‎(Leica Ultracut UCT, Tokyo, Japan) ‎and stained with 1% lead acetate. Sections were examined under JEOL-JEM-2100F TEM ‎(JEOL 1011 CX, Tokyo, Japan) ‎operating at 200 kV.

### 4.3. Statistical Analysis

Analysis of Variance (ANOVA) was performed across the ‎groups with or Bonferroni Post-Hoc test. Significance was set at *p* ≤ 0.05 and all statistical analyses were carried out using SPSS for ‎Windows (version 16.0, Chicago, IL, USA).‎

## 5. Conclusions

In summary, this study demonstrated that in mice, the skeletal muscle mitochondrial ‎biogenesis and oxidative metabolism can be severely affected by HFD, and vitamin D treatment ‎restored associated histological and ultrastructural abnormalities. Additional studies are ‎warranted to elucidate the molecular mechanism(s) by which vitamin D emulates mitochondrial ‎degeneration under various dietary conditions.‎ 
